# Hepatoprotective natural polyphenol extracts: molecular mechanisms, stabilization-delivery strategies, and translational bottlenecks for functional food applications

**DOI:** 10.3389/fimmu.2026.1988758

**Published:** 2026-10-23

**Authors:** Xiaoxiao Yao, Ruining Zhang, Yi Wang, Yi’en Xiang

**Affiliations:** 1 Jilin University Engineering Laboratory for Translational Medicine of Hepatobiliary and Pancreatic Diseases, The Second Norman Bethune Clinical Medical College of Jilin University, Changchun, China; 2 College of Food Science and Engineering, Jilin Agricultural University, Changchun, China

**Keywords:** hepatoprotection, immunometabolism, liver immunity, multi-target mechanism, polyphenols, stabilization-delivery strategy

## Abstract

Natural polyphenols exert multi-target hepatoprotective activities via orchestrating hepatic redox homeostasis, lipid metabolism, and hepatic innate-adaptive immune responses, yet their clinical and food-grade translation is hampered by poor stability, low bioavailability and dose-dependent safety risks. This review integrates polyphenol classification with critical toxicological analysis to address pro-oxidant, endocrine-disrupting, and immune-perturbing hazards under high-dose supplementation. We systematically construct multipathway hepatoprotective networks, highlighting polyphenol-driven immunomodulatory effects including macrophage polarization, NLRP3 inflammasome repression, and immunometabolic reprogramming, alongside classical antioxidant, anti-inflammatory, lipid-homeostasis-regulating, anti-pyroptotic, anti-apoptotic and antifibrotic outputs. Four stabilization-delivery approaches are assessed, alongside key bottlenecks including crude extract batch variation, incomplete recognition of gut-liver immune crosstalk, costly nanocarriers and deficient quantitative immune-phenotyping evaluation systems. An integrated strategy combining molecular modification, green stabilization, hepatocyte-/immune-cell-responsive delivery and therapeutic-efficacy traceability is proposed to advance polyphenol-based hepatoprotective candidates for immune-mediated liver injury.

## 1 Introduction

The liver serves as the central organ for metabolism, detoxification, and immune regulation in the body. The global epidemiological landscape of liver diseases has undergone significant shifts in recent years. While considerable progress has been made in the prevention and control of traditional viral hepatitis, the prevalence of metabolism-associated liver diseases has been rising steadily and affecting younger populations, posing a growing global public health burden (1). Epidemiological data indicate that the global prevalence of non-alcoholic fatty liver disease (MASLD) among adults reached 30% in 2023 (2–4). Moreover, predictive models estimate that by 2050, the number of MASLD patients will reach approximately 1.8 billion, representing a 42% increase from 2023, with a standardized prevalence rate of 15,774.9 per 100,000 and an average annual growth rate of 0.3% (5).

Accumulating evidence has established that most chronic liver injuries, including MASLD/NASH, alcoholic hepatitis, and toxin-provoked hepatic damage, are essentially immune-metabolic disorders (6, 7). Liver resident immune cells (Kupffer cells, hepatic NK/NKT cells) and infiltrating peripheral immune cells (macrophages, neutrophils, T-cell subsets) act as central mediators that bridge metabolic disturbance, oxidative stress, and progressive tissue fibrosis (8, 9). Dysregulated innate immune signaling, exemplified by TLR4-MyD88 cascades and NLRP3 inflammasome activation, drives the release of damage-associated molecular patterns (DAMPs) and pro-inflammatory mediators to amplify hepatic pathological deterioration (10, 11).

Natural bioactive phytochemicals have garnered extensive attention for their high biosafety, low toxicity, and multi-target synergistic regulatory properties, serving as ideal candidate resources for hepatoprotective functional foods and adjuvant therapies (12, 13). These natural components can ameliorate hepatic lesions by modulating multiple core signaling pathways including Bax/Bcl-2 and NF-κB, thereby regulating hepatocellular oxidative stress, inhibiting inflammatory cascade responses, and correcting hepatic lipid metabolic disorders (14). Among numerous natural active ingredients, polyphenols are recognized as one of the most well-documented and biologically potent functional components for liver protection. As the largest subclass of polyphenols, flavonoids possess superior hepatoprotective activity and have become the core research focus of natural liver-protective products (15). Widely distributed in medicinal and edible plants, fresh fruits, vegetables, and plant extracts, flavonoids are characterized by unique phenolic hydroxyl structures. This structural feature enables them to scavenge excess intracellular free radicals, mitigate oxidative damage to hepatocytes, suppress hepatocellular apoptosis, regulate hepatic inflammatory pathways, modulating the gut microbiota, maintaining the integrity of barrier function, and improve lipid metabolism and detoxification function (16–18). Through the above multi-dimensional regulatory mechanisms, flavonoids effectively block the progressive deterioration of liver lesions and alleviate multiple pathological injuries, including fatty liver degeneration and chemical-induced hepatic damage (19, 20). Compared with single-target synthetic chemical drugs, polyphenolic and flavonoid components exhibit prominent advantages of low toxic side effects, multi-pathway regulatory effects, and broad applicability, demonstrating great theoretical research value and industrial transformation prospects in daily liver health maintenance, auxiliary repair of liver injury, and early intervention of metabolic liver diseases.

Although polyphenols exert multi-target hepatoprotective benefits, inherent physicochemical drawbacks such as poor stability create substantial technical bottlenecks for the research and industrial translation of polyphenol-based liver-protective products. Current molecular mechanistic studies of polyphenol-mediated liver protection are relatively scattered and lack systematic and in-depth theoretical frameworks. In addition, most polyphenolic active ingredients are sensitive to light, heat, and pH fluctuations, leading to severe activity attenuation during processing, transportation, and storage. Meanwhile, their inherent low oral bioavailability and poor liver-targeted delivery efficiency greatly limit their *in vivo* hepatoprotective efficacy (21).

While numerous reviews have summarized polyphenols’ antioxidant, lipid-regulatory, and anti-fibrotic properties, few have systematically consolidated polyphenol-mediated immunomodulatory circuits linking gut-liver immune crosstalk to hepatoprotection. Against this backdrop, this review systematically summarizes the molecular mechanisms of typical natural polyphenolic bioactive extracts in liver protection, emphatically analyzes the key technical bottlenecks restricting their stability and liver-targeted delivery performance, and comprehensively reviews the latest research progress in structural modification, stability optimization, and controlled-release delivery strategies of polyphenols. This study aims to provide a systematic theoretical basis for dissecting polyphenol-centered hepatic immunoregulation, as well as for further development, clinical popularization, and industrial transformation of high-efficiency natural hepatoprotective functional products.

## 2 Types of liver injury and core pathological pathways

### 2.1 Major types of liver injury and their pathological features

In current global research, common liver injuries can be categorized into four major types based on etiological factors and pathological characteristics: metabolic liver injury, alcoholic liver injury, chemical liver injury, and hepatic fibrotic injury (22). Metabolic liver injury currently has the highest global incidence among all types of liver injury, with MASLD and its progressive form, non-alcoholic steatohepatitis (NASH), as the core representatives (23). These conditions are primarily induced by high-fat and high-sugar diets, metabolic disorders, obesity, and related factors. Their core pathological features include abnormal hepatic lipid accumulation, hepatocellular ballooning, inflammatory cell infiltration, and fibrotic remodeling of liver tissue. The disease course is characterized by insidious onset and gradual progression, representing a major cause of chronic liver disease in young and middle-aged adults worldwide. Immunologically, progressive NASH is characterized by Kupffer-cell activation, M1-type macrophage polarization, massive neutrophil and CD4^+^/CD8^+^ T-cell infiltration, and NLRP3 inflammasome hyperactivation, which fuels inflammatory amplification and fibrogenesis (8, 24). Alcoholic liver injury is mediated by chronic excessive alcohol consumption, with the pathological progression sequentially manifesting as alcoholic fatty liver, alcoholic hepatitis, hepatic fibrosis, and cirrhosis (25). The core injury mechanism involves hepatocytotoxic damage caused by the accumulation of acetaldehyde, a metabolite of alcohol, accompanied by oxidative stress bursts and persistent hepatic inflammatory responses (26). Ethanol-derived intestinal barrier disruption facilitates microbial-associated molecular patterns (PAMPs) translocation into portal blood, triggering robust hepatic innate-immune activation via TLR-family receptors (27, 28). Chemical liver injury is primarily triggered by exogenous toxicants such as drugs, environmental toxins, heavy metals, and food additives. It is characterized by acute onset and severe damage, manifesting principally as acute hepatocellular necrosis and a sharp elevation in liver function indicators, and constitutes a major cause of clinical acute liver injury (29, 30). Massive hepatocyte necrosis releases abundant DAMPs, which recruit circulating immune cells and ignite sterile hepatic inflammatory responses (31, 32). Hepatic fibrosis represents the common terminal pathological alteration shared by various acute and chronic liver injuries, with aberrant activation of hepatic stellate cells and excessive deposition of extracellular matrix as its core features. Persistent fibrotic progression disrupts the normal structural architecture and function of liver tissue, ultimately leading to irreversible cirrhosis and hepatocellular carcinoma (33). Immune-cell-derived cytokine milieus (TGF-β, TNF-α, IL-1β) are critical upstream triggers for hepatic stellate-cell trans-differentiation toward myofibroblasts. Triggered by various injurious stimuli, oxidative stress, dysregulated immune-cell responses, and lipid metabolic disorders synergistically induce sequential hepatic steatosis, steatohepatitis and liver fibrosis, which ultimately progress to cirrhosis. **Figure 1** intuitively depicts the evolution of liver injury, highlighting immune-cell participation and its key molecular mechanisms.

**Figure 1:**
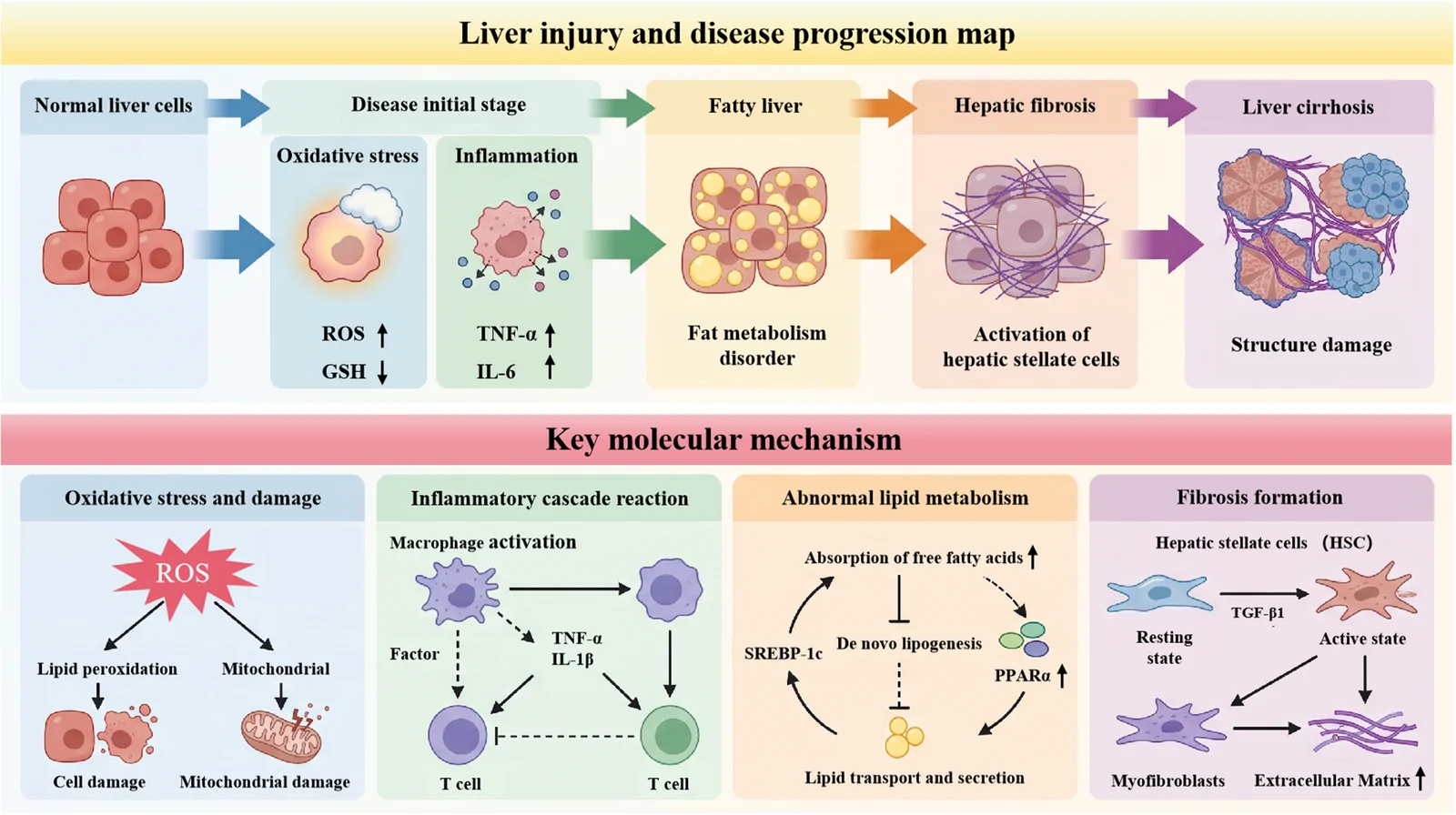
The image illustrates the progressive pathological changes from normal hepatocytes to simple fatty liver, hepatic fibrosis, and liver cirrhosis upon chronic liver injury. Four core pathological events are highlighted, including oxidative damage, macrophage-mediated inflammatory response, disordered hepatic lipid metabolism, and fibrogenesis triggered by activated hepatic stellate cells.

### 2.2 Core pathological pathways regulating liver injury

Although the pathological progression of different types of liver injury exhibits mutual overlap and highly coupled pathways, oxidative stress, inflammatory dysregulation, lipid metabolic imbalance, and aberrant apoptosis represent the core common pathological mechanisms underlying various forms of liver injury. These mechanisms correspond to multiple classical regulatory signaling pathways, which also serve as key targets for the hepatoprotective effects of various therapeutic agents (34, 35). The activation intensity of these pathways varies considerably across different injury stages, as well as among distinct cell subtypes including hepatocytes, hepatic stellate cells, and immune cells, and their interplay mutually amplifies the overall damage effects. Collectively, these mechanisms can be categorized into four core pathological modules: cell death, fibrosis, stress responses, and inflammation, with positive feedforward cycles operating among these modules. Oxidative stress induces hepatocyte apoptosis and necrosis, and the necrotic cells release damage-associated molecular patterns (DAMPs), which are sensed by pattern-recognition receptors on Kupffer cells and infiltrating macrophages to activate downstream innate-inflammatory pathways including the NLRP3 inflammasome (32). Persistent chronic inflammation further stimulates hepatic stellate cell activation and accelerates extracellular matrix deposition, thereby promoting fibrotic progression. In turn, fibrosis exacerbates hepatic microcirculatory disturbances, which reciprocally aggravate oxidative stress and cell death (36). These four processes drive one another in a vicious cycle, collectively determining the overall progression of liver injury from mild inflammation and steatosis to irreversible cirrhosis and even hepatocellular carcinoma. These pathways are interconnected and synergistically regulate the progression of liver injury, constituting a molecular network system underlying hepatic pathological damage. As summarized in **Table 1**, we have systematically outlined the core pathways and key functional proteins corresponding to each module.

**Table 1:** Core regulatory pathological pathways of liver injury.

Pathological process	Core regulatory pathway	Key proteins/molecules	Primary function	References
Cell Death & Injury	Ferroptosis Pathway	GSH-PX, SREBP1-c,	Lipid peroxidation-driven programmed cell death, critical in ischemia-reperfusion injury, etc.	(37)
	Integrated Stress Response (ISR)	PERK/eIF2α/ATF4/CHOP	Perceives cellular stress; overactivation exacerbates acute liver failure via mitochondrial dysfunction and inflammation.	(38)
	MAPK Signaling Pathway	ASK1, MKK4, JNK, p38- MAPK	Transmits death signals; its activation is closely linked to hepatocyte necrosis and apoptosis in various liver injuries.	(39)
Inflammatory & Immune Response	NLRP3 Inflammasome Pathway	NLRP3, ASC, Caspase-1, GSDMD, IL-1β	Innate-immune sensor; mediates pyroptosis and maturation of pro-inflammatory IL-1β/IL-18; central driver of NASH, alcoholic hepatitis and toxic liver injury	(10)
Fibrosis & Repair	TGF-β Signaling Pathway	TGF-β1, SMAD, α-SMA	Core pro-fibrotic pathway. Activates hepatic stellate cells, promotes massive collagen production, leading to ECM deposition.	(40, 41)
Cellular Stress	Endoplasmic Reticulum Stress (ERS) Pathway	PERK, IRE1α, ATF6, GRP78, CHOP	Shifts from protective adaptation to pro-apoptotic signaling upon protein overload, involved in multiple liver diseases.	(42, 43)
	Keap1-Nrf2/HO-1 Pathway	Keap1, Nrf2, HO-1	Core antioxidant pathway. Scavenges ROS upon activation, a key defense mechanism against oxidative stress in the liver.	(44)
Inflammation & Immunity	NF-κB Signaling Pathway	NF-κB, TNF-α, IL-6	The “master switch” of inflammatory responses; regulates transcription of numerous pro-inflammatory cytokines, mediating hepatic inflammatory injury.	(45)
	YAP1/Nrf2 Pathway	TRIM16, YAP1, Nrf2	E3 ubiquitin ligase TRIM16 activates Nrf2 by stabilizing YAP1, exerting anti-inflammatory and antioxidant protective effects.	(46)

Glutathione peroxidase, GSH-PX; Sterol regulatory element-binding protein 1c, SREBP1-c; Protein kinase RNA-like endoplasmic reticulum kinase, PERK; Eukaryotic translation initiation factor 2 alpha, eIF2α; Activating transcription factor 4, ATF4; C/EBP homologous protein, CHOP; Apoptosis signal-regulating kinase 1, ASK1; Mitogen-activated protein kinase kinase 4, MKK4; c-Jun N-terminal kinase, JNK; p38 mitogen-activated protein kinase, p38-MAPK; NOD-like receptor family pyrin domain-containing 3, NLRP3; Apoptosis-associated speck-like protein containing a CARD, ASC; Cysteinyl aspartate specific proteinase-1, Caspase-1; Gasdermin D, GSDMD; Interleukin-1 beta, IL-1β; Transforming growth factor beta 1, TGF-β1; Sma- and Mad-related protein, SMAD; Alpha-smooth muscle actin, α-SMA; Inositol-requiring enzyme 1 alpha, IRE1α; Activating transcription factor 6, ATF6; 78 kDa glucose-regulated protein, GRP78; Kelch-like ECH-associated protein 1, Keap1; Nuclear factor erythroid 2-related factor 2, Nrf2; Heme oxygenase 1, HO-1; Nuclear factor kappa B, NF-κB; Tumor necrosis factor alpha, TNF-α; Interleukin-6, IL-6; Tripartite motif-containing protein 16, TRIM16; Yes-associated protein 1, YAP1.

Although pharmacological adjunctive therapies targeting these core pathological pathways of liver injury are currently available, conventional clinical chemical drugs, hormonal agents, and hepatoprotective formulations generally suffer from inherent limitations, including single-target mechanisms, poor long-term safety profiles, difficulty in reversing chronic and end-stage liver injury, and the capacity to provide only symptomatic intervention without achieving sustained protective effects. Consequently, natural plant-derived bioactive extracts, owing to their unique advantages of low toxicity, high safety, multi-target synergistic regulation, and the ability to integrate both prevention and adjunctive therapy, can effectively address the deficiencies of traditional therapeutic systems and demonstrate excellent research and application potential.

Collectively, oxidative stress, dysregulated immune signaling, lipid dysregulation, apoptosis and pyroptosis constitute common pathological drivers across multiple liver-injury subtypes. Natural polyphenols can interfere with these overlapping pathological nodes, especially by reshaping hepatic immune homeostasis, which forms the theoretical basis for their multi-target hepatoprotective capacity.

## 3 Introduction to natural polyphenols and toxicological evaluation

### 3.1 Natural polyphenolic active substances

Natural active extracts refer to a class of functionally active substances with defined structures and significant biological activities, which are isolated and purified from natural biomass sources such as plants, microorganisms, and algae through physical, chemical, or biological extraction techniques. They constitute the core effective fraction through which natural products exert their pharmacological activities and nutritional regulatory functions (47–49). Distinct from chemically synthesized monomeric drugs, natural bioactive substances are characterized by structural diversity, low toxicity, good biocompatibility, and multi-target regulatory properties. The majority are derived from medicine-food homology resources, possessing both dietary safety and pharmacological efficacy, making them high-quality resources for the development of novel hepatoprotective functional formulations and for achieving dietary intervention and adjunctive therapy in liver diseases (50). Polyphenolic natural products are the most widely distributed and largest class of secondary metabolites in the plant kingdom, which are chemically defined as aromatic compounds containing two or more phenolic hydroxyl groups in their molecular structures (51). Based on differences in chemical structures and physicochemical properties, natural polyphenolic active ingredients with confirmed definite hepatoprotective effects can be classified into flavonoids and non-flavonoid polyphenols. Differences in component structures directly determine their distinctions in the focus of hepatoprotective activity, action intensity and physicochemical stability (52).

#### 3.1.1 Flavonoids

Flavonoids are polyphenolic secondary metabolites widely distributed in medicinal and edible homologous plants, featuring a core C6-C3-C6 diphenyl three-carbon benzopyran scaffold with multiple phenolic hydroxyl groups on the molecular structure. They are classified into subcategories including flavones, flavonols, anthocyanins, flavanols and isoflavones based on differences in the saturation degree, double bonds and glycosyl substitution of the C-ring, with representative bioactive monomers such as quercetin, rutin, luteolin and silymarin (53). Free flavonoids exhibit high lipophilicity and poor water solubility, while glycosylated flavonoids possess markedly improved water solubility owing to conjugated hydrophilic glycosyl groups. The conjugated phenolic hydroxyl groups endow flavonoids with potent antioxidant capacity; nevertheless, they are susceptible to oxidative degradation under light, high temperature and pH fluctuations, and generally taste bitter and astringent. Flavonoids exert multi-target hepatoprotective effects via metal ion chelation, activation of the Keap1-Nrf2-ARE signaling pathway and inhibition of the NF-κB inflammatory cascade, making them the most extensively investigated small-molecule natural hepatoprotective constituents to date (52).

#### 3.1.2 Polyphenols(non-flavonoid polyphenols)

Polyphenols are a broad class of plant secondary metabolites containing two or more phenolic hydroxyl groups directly attached to aromatic rings. Herein, they specifically refer to polyphenol subclasses excluding flavonoids, which lack a unified parent skeleton and are mainly divided into three major categories: phenolic acids (chlorogenic acid, gallic acid), stilbenes (resveratrol), and condensed/hydrolyzable tannins, featuring abundant conjugated aromatic structures in their molecules. Most polyphenols show moderate water solubility, whereas the water solubility of condensed tannins declines with increasing polymerization degree (51). Their phenolic hydroxyl groups confer powerful free radical scavenging capacity, yet they exhibit extremely poor stability against heat, oxygen and light, leading to severe loss of bioactivity during processing and storage. Sensory-wise, they possess intense bitter and astringent tastes and tend to form complexes and precipitates with proteins. They simultaneously block oxidative stress, suppress the MAPK/NF-κB inflammatory pathways and alleviate hepatic lipid accumulation, and thus are applicable to the intervention of alcoholic and metabolic liver injuries (54).

As shown in **Figure 2**, polyphenolic compounds with hepatoprotective activities and their sources are summarized. In contrast to the single-target action mode of chemical drugs, polyphenols can form a multi-target cross-regulated network covering five major pathological modules: oxidative stress, inflammation, lipid metabolism, apoptosis and fibrosis (55).

**Figure 2:**
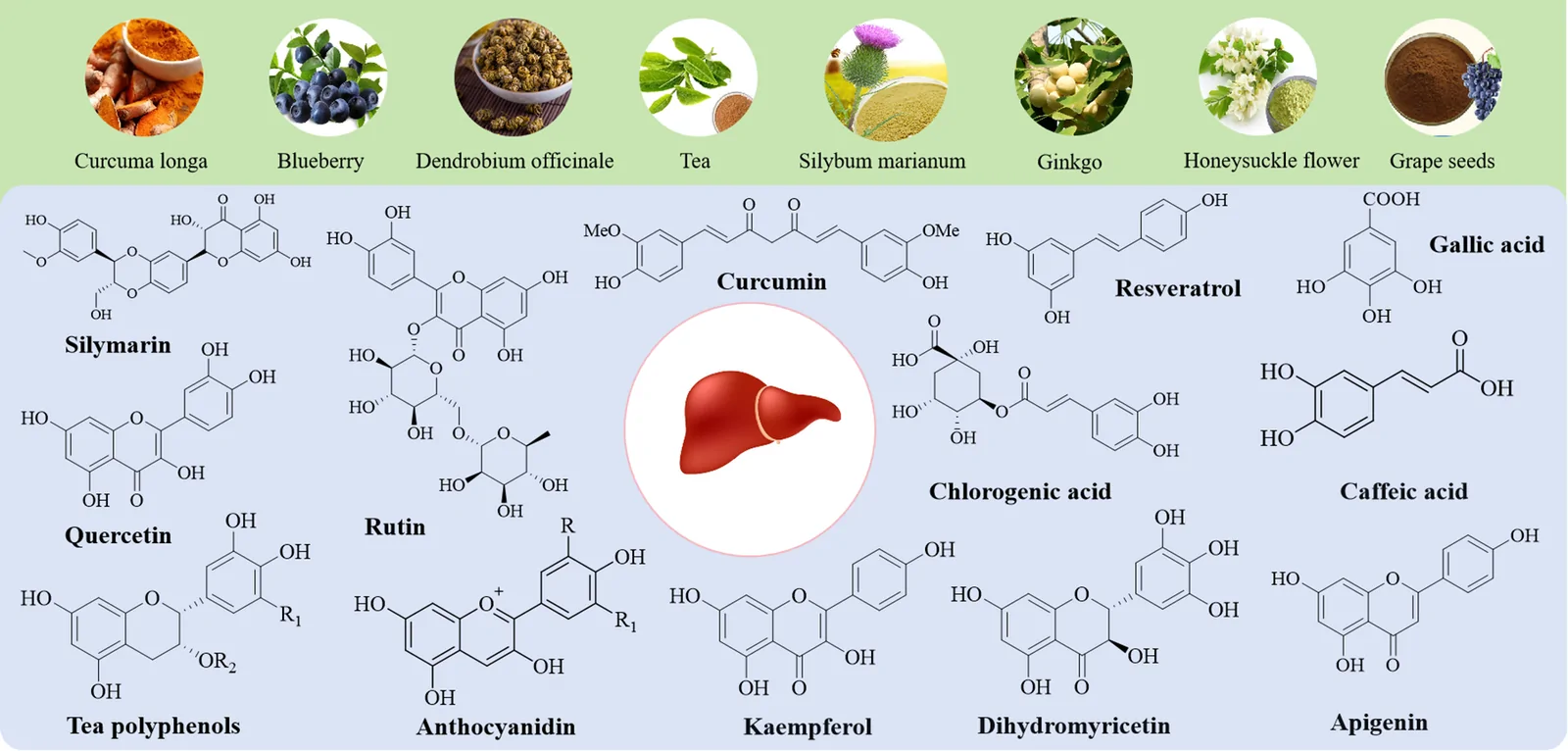
Summary of typical polyphenol-rich natural plants with proven hepatoprotective activities, alongside the chemical structures of their representative bioactive flavonoid and non-flavonoid polyphenol constituents.

### 3.2 Toxicological considerations of polyphenols

Dietary polyphenols and flavonoids exhibit a classic hormetic dose-response relationship, wherein low to moderate intakes confer antioxidant and hepatoprotective benefits, whereas high-dose exposure may provoke pro-oxidant, cytotoxic, and even genotoxic effects (56). This “redox paradox” arises from the intrinsic chemical reactivity of polyphenolic structures: at elevated concentrations, cytochrome P450-mediated oxidation generates semiquinone radicals and reactive ortho-quinones, which undergo redox cycling to produce superoxide and hydrogen peroxide, deplete glutathione, and covalently bind to protein thiols (57, 58). Consequently, the same phenolic hydroxyl groups that scavenge radicals at physiological levels become sources of oxidative stress when present in excess, necessitating a careful dose consideration in functional food development.

Hepatotoxicity has been documented for several representative polyphenols at supraphysiological doses. Quercetin, at intraperitoneal doses ≥20 mg/kg in rats, induced dose-related structural alterations in liver tissues even when serum transaminases remained normal, indicating that routine biomarkers may underestimate early damage (57). Nevertheless, it should be noted that intraperitoneal injection bypasses gastrointestinal absorption and hepatic first-pass metabolism. Accordingly, the pharmacological effects observed via injection cannot represent the outcomes under oral administration. Researchers ought to compare oral exposure with intraperitoneal injection to identify appropriate administration routes and corresponding dosage requirements. Epigallocatechin-3-gallate (EGCG), the major green tea catechin, is the most extensively studied case: high-dose EGCG-induced hepatotoxicity is associated with increased markers of oxidative stress, including hepatic lipid peroxidation and elevated plasma 8-isoprostane (59), and the European Food Safety Authority (EFSA) has concluded that daily intake exceeding 800 mg of total catechins from supplements raises a clear risk of hepatocellular injury (60). Curcumin supplements with enhanced bioavailability have been linked to several case reports of drug-induced liver injury. One such report describes a case of a 40-year-old female with increasing fatigue, pruritus, and dark urine following consumption of turmeric and black pepper “wellness shots”, leading to a significant drug-induced liver injury (61). These examples underscore that the safety margin of each polyphenol is highly compound-specific and dose-dependent. However, these cases do not negate the hepatoprotective value of polyphenols; instead, they highlight the urgency of establishing individualized safe dosage standards. Given the substantial variation in polyphenol bioavailability, improper doses may trigger adverse effects. Acknowledging this avoids one-sided assertions and renders discussions on relevant mechanisms and translational research more objective.

In summary, current studies on dietary polyphenols exhibit notable limitations, with a prevalent research bias toward efficacy development at the expense of safety analysis. While the safety of polyphenols at regular dietary intakes is well-recognized, quantitative safety thresholds for their application in high-dose functional foods and dietary supplements remain undefined. Their potential pro-oxidant and endocrine-disrupting risks, as well as long-term safety, still lack systematic validation. Most existing studies one-sidedly focus on the hepatoprotective activities of polyphenols but overlook their bidirectional dose-dependent effects. Moreover, the modulatory effects of formulation structure, food matrix and individual physiological status on their pharmacodynamic and toxicological profiles have not been elucidated, leaving the balance between hepatoprotective benefits and safety risks poorly understood and severely hindering their industrial translation.

## 4 Molecular mechanisms of hepatoprotection by polyphenols

Given these research gaps, toxicological assessment alone cannot overcome translational bottlenecks. It is imperative to decipher the core hepatoprotective mechanisms at the molecular level. Systematically unraveling the key pathways and targets by which polyphenols modulate hepatic oxidative stress, inflammation, lipid homeostasis, apoptosis and hepatic fibrosis enables discrimination between their pharmacological benefits and toxic insults, facilitating accurate definition of their safe action window. Accordingly, this chapter systematically elaborates the core molecular mechanisms underlying polyphenol-mediated hepatoprotection and constructs an integrated regulatory network for polyphenol-derived liver protection.

A substantial body of research has demonstrated that polyphenols can exert multidimensional hepatoprotective effects through molecular interactions with the aforementioned core targets, including protein binding, transcriptional regulation, and pathway activation or inhibition. We have systematically organized the core molecular mechanisms of hepatoprotection by natural bioactive substances according to their associated pathological pathways. The representative molecular mechanisms of hepatoprotection by natural bioactive extracts are summarized in **Table 2**, and can be categorized into the following types.

**Table 2:** The molecular mechanism of liver protection by representative natural active extracts.

Natural plants	Active components	Hepatoprotective mechanisms	References
*Desmodium heterocarpon* (L.)	Flavonoids	Activate the Keap1/Nrf2 pathway, promote the nuclear translocation of Nrf2, reduce ROS, and protect mitochondrial function	(62)
*Apocynum venetum* L.	Flavonoids	Activate the AMPK and NRF2 pathways, improve lipid metabolism and alleviate oxidative stress	(63)
*Lonicera caerulea* L.	Polyphenols	LCP alleviates LPS-induced liver injury by suppressing the nuclear translocation of NF-kappa B p65 and activation of the MAPK signaling pathway	(64)
*Curcuma longa*	Curcumin	Decreased oxidative stress-associated protein expression, mainly involving CYP2E/Nrf2/ROS signaling pathway	(65)
*Polygonum perfoliatum* L.	Flavonoids	The levels of TNF-α, IL-6 and Hsp90 in the mice serum treated by flavonoids were all reduced	(66)
–	Quercetin	Quercetin could elevate the expression of nuclear factor E2-related factor 2(Nrf2)/HO-1 and ameliorate ethanol-induced acute liver injury in rats	(67)
*Glycyrrhiza uralensis* Fisch.	Flavonoids	Alleviate liver injury in mice by activating the Nrf2 pathway	(68)
Grape Seed	Polyphenols	Grape seed extract can effectively reduce related pathological processes by regulating the TLR4/NF-κB and Nrf2/HO-1 axes	(69)
*Chrysanthemum morifolium* Ramat.	Flavonoids	Activate PPARα/γ/δ, regulate lipid metabolism and alleviate inflammation and fibrosis	(70)
*Chaihu-Baishao*	Kaempferol	Targeted inhibition of PTGS2 (COX-2) and reprogramming of macrophage polarization	(71)
*Hibiscus manihot* L.	Flavonoids	Regulate the intestinal flora and inhibit the TLR2/MAPK pathway	(72)
*Siraitia grosvenorii*	Flavonoid Glycoside	Inhibit the TLR4/NF-κB/MyD88 pathway; Activate the Nrf2/HO-1 pathway	(73)
*Nigella sativa*	–	Down-regulate the expression of NF-κB, TNF-α, IL-1β, and COX-2 genes	(74)
*Ocimum gratissimum*	polyphenol extract (OGPE)	OGPE (12 mg/kg BW) maintained the levels of serum alanine aminotransferase and aspartate aminotransferase, as well as the levels of malondialdehyde, catalase and a-smooth muscle actin in liver tissues from CCl4-induced changes	(75)
*Aronia melanocarpa*	Polyphenols	Inhibit the expression of inflammatory factors IL-6, IL-1β, TNF-α and their corresponding mRNAs	(54)
*Curcuma longa*	Curcumin	Curcumin can reduce the levels of serum cytokines, including IL-1β, IL-6, and TNF-α	(65)
White peony	polyphenols	White peony polyphenols reduced the serum levels of IL-6, IL-12, TNF-α, and IFN-γ in mice with liver injury https://doi.org/10.3390/antiox8110524	(76)
*Dark tea*	Polyphenols	Inhibition of weight gain, reduction of lipid dysfunction	(77)
*Dianthus superbus* L.	Flavone C-Glycosides	Upregulation of PPARγ promotes lipid metabolism, enhances INSR/PI3K/AKT insulin signaling, and improves MASLD	(78)
*Clinacanthus nutans* leaf	Tannin	Prevent the progression of simple fatty liver to fatty liver inflammation (MASH) through antioxidant mechanisms	(79)
Holly	Polyphenols	Holly polyphenols supplementation reduced plasma and/or liver TNF-α, IL-6 and IL-1β levels, and liver mRNA expression of IL-6, TNF-α and COX2	(80)
‘Limoncella’ Apple	Polyphenols	LAPE significantly improved the serum lipid peroxidation and liver injury in DNBS-induced colitis, as well as reduced the nuclear transcription factor-kappaB activation	(81)
Triadica cochinchinensis honey	Polyphenols	Polyphenols can restore the levels of ALT and AST, and TCH intervention can significantly improve the pathological changes of liver tissue in alcohol-exposed mice	(82)
*Black Raspberry*	Polyphenols	Significantly alleviated obesity, insulin resistance, lipid and glucose metabolism disorders, as well as liver damage caused by a high-fat diet	(83)
*Scutellaria barbata*	Alkaloids, flavonoids, and polysaccharides	Down-regulate cleaved-Caspase-3 and Bax, up-regulate BCL-2, restore mitochondrial membrane potential, and inhibit hepatocyte apoptosis	(84)
Tea	Polyphenols	TP protects against hepatic I/R-induced injury by inhibiting I/R-induced oxidative damage and liver cell apoptosis	(85)
Crocus sativus (saffron)	Flavonol glycosides	Activate the Nrf2/HO-1 signaling pathway, and inhibit oxidative stress and cell apoptosis	(86)
Fenugreek	Quercetin and kaempferol	Fenugreek extract ameliorated liver inflammatory and fibrogenic re-sponses through miR-146a/miR-29b/TGF-8/Smad axis	(87)
*Cranberry*	Flavonoids	The hepatoprotective potential of cranberry flavonoids could be due to specific prevention of rat liver mitochondrial damage	(88)
*Silybum Marianum* (Milk Thistle)	Silymarin	Suppression and downregulation of Collagen I, TGF-β, SMAD-3, and MMP-3 and upregulation of the hepatic progenitor stem cells markers HNF1α, FOXl1, CD90, Vimentin, and CD105	(89)
*Dendrobium chrysotoxum* Lindl.	Erianin	Attenuate CCl4-and TGF-β 1-induced HSCs activation and liver fibrosis	(90)

### 4.1 Molecular mechanisms of anti-oxidative stress

Under oxidative stress conditions, bioactive components derived from plants can activate the Keap1-Nrf2-ARE antioxidant signaling pathway (62). Through covalent modification of the Keap1 protein or by promoting its conformational changes, Nrf2 is released from Keap1-mediated inhibition and translocates into the nucleus, where it binds to the antioxidant response element (ARE) and initiates the transcription of a series of downstream antioxidant enzymes (such as SOD, CAT, and GPx) and phase II detoxifying enzymes (such as HO-1), thereby efficiently scavenging reactive oxygen species (ROS) and restoring redox homeostasis within hepatocytes (91). Concurrently, these bioactive components can also suppress the NF-κB inflammatory pathway, reducing the release of pro-inflammatory cytokines such as TNF-α and IL-6, thus breaking the vicious cycle between oxidative stress and inflammation (54, 92). By activating the AMPK pathway, they regulate lipid metabolism and reduce fatty acid synthesis, thereby diminishing the substrate sources of oxidative stress at the source. Furthermore, they block hepatocyte apoptosis by modulating the Bcl-2/Bax balance and inhibiting Caspase activation (63, 92). Ultimately, these four pathways intertwine and reinforce one another, collectively constructing a comprehensive molecular barrier that protects hepatocytes through an “anti-oxidation–anti-inflammation–lipid regulation–anti-apoptosis” integrated network.

Notably, the Keap1-Nrf2-ARE pathway operates not only inside hepatocytes but also within hepatic immune cells. Nrf2 activation in Kupffer cells constrains pro-inflammatory M1 polarization and limits NLRP3 inflammasome assembly, connecting cellular antioxidant defense to hepatic immune homeostasis (93, 94).

### 4.2 Immunomodulatory and anti-inflammatory hepatoprotective mechanisms

The core molecular mechanism by which polyphenols exert anti-inflammatory hepatoprotective effects is a multi-target network centered on the inhibition of the NF-κB signaling pathway, while simultaneously coordinating the regulation of multiple inflammation-related pathways (95). Under inflammatory stimulation, polyphenols interfere with upstream signals such as TLR4/MyD88 to suppress the activation of IκB kinase, thereby blocking the nuclear translocation of NF-κB and downregulating the transcriptional levels of pro-inflammatory cytokines including TNF-α, IL-6, and IL-1β, thus curbing the inflammatory burst at its source (54). Concurrently, these components also inhibit the phosphorylation of the MAPK family (such as p38 and JNK), blocking the cascade amplification of inflammatory signals. In addition, polyphenols can synergistically ameliorate lipid metabolic disorders, alleviate inflammation and fibrosis by activating PPARs nuclear receptors, and scavenge reactive oxygen species through the activation of the Nrf2/HO-1 antioxidant pathway. Given that oxidative stress and inflammation are mutually causal, this mechanism indirectly attenuates the persistent driving factors of the inflammatory response as well (73).

Apart from canonical NF-κB and MAPK cascade inhibition, polyphenols exert prominent immunomodulatory functions by reshaping hepatic immune-cell phenotypes. Polyphenols can suppress M1-proinflammatory polarization and promote anti-inflammatory M2-type polarization of Kupffer-derived macrophages, decreasing the local hepatic pro-inflammatory cytokine milieu (96). Multiple polyphenol monomers are capable of restraining NLRP3 inflammasome assembly, repressing caspase-1 cleavage and subsequent gasdermin-D (GSDMD)-driven pyroptosis, a lytic inflammatory cell death mode amplifying liver sterile injury (97, 98). In terms of adaptive immunity, polyphenols modulate CD4^+^ T-helper subset differentiation, curbing pathogenic Th1/Th17 responses while expanding protective T-regulatory (Treg) populations. Given that oxidative stress and inflammation are mutually causal, this mechanism indirectly attenuates the persistent driving factors of the inflammatory response as well (99). Ultimately, these pathways intertwine and reinforce one another, constructing a full-chain anti-inflammatory hepatoprotective molecular barrier that spans from inhibiting the initiation of inflammation and blocking signal transduction to eliminating inflammatory triggers. A studie have demonstrated that apple polyphenol extract mitigates cholestatic liver injury via SIRT1-dependent pathways, regulating bile acid metabolism and intestinal barrier function in a sex-specific fashion, where estrogen receptor signaling may mediate these differential hepatoprotective effects (132). Another study indicated that quercetin, a naturally safe flavonoid, can regulate inflammatory responses through SIRT1-dependent NF-κB and NLRP3 signaling pathways, and such SIRT1-mediated immunomodulatory activity may contribute to its hepatoprotective capacity against aging-related tissue injury (133).

### 4.3 Molecular mechanisms of regulating lipid metabolic disorders

Polyphenols exert synergistic regulatory effects on lipid metabolism through a network involving multiple key signaling pathways that balance lipid synthesis and oxidative catabolism. These bioactive substances can inhibit the activity of SREBP-1c and its downstream key lipogenic enzymes, such as FAS and ACC, thereby reducing hepatic *de novo* lipogenesis (DNL) (100, 101). Simultaneously, they activate PPARα and its downstream key proteins involved in fatty acid oxidation, such as CPT-1, which enhances mitochondrial β-oxidation of fatty acids and promotes lipid clearance. AMPK, serving as the core energy metabolism sensor, upon activation, can both suppress SREBP-1c-mediated anabolic pathways and promote fatty acid oxidation (102). In addition, certain extracts can indirectly regulate hepatic lipid metabolism through other pathways, including upregulating PPARγ expression to promote lipid metabolism, enhancing insulin signaling pathways (such as PI3K/AKT) to ameliorate insulin resistance, and modulating gut microbiota and their metabolites (e.g., O-Nornuciferine) (103). Ultimately, these pathways intertwine to construct a comprehensive lipid metabolism regulatory network that spans the full chain from “reducing lipid synthesis—promoting lipid catabolism—improving energy metabolism”. Of note, lipid metabolic reprogramming also occurs within liver immune cells. Excessive lipid deposition fuels pro-inflammatory macrophage glycolysis and inflammasome priming. Polyphenols ameliorate immunometabolic dysfunction of intrahepatic immune populations beyond merely correcting hepatocyte steatosis, partially breaking the crosstalk between metabolic disturbance and immune overactivation (6, 104).

### 4.4 Molecular mechanisms inhibiting hepatocyte apoptosis and inflammatory pyroptosis

Polyphenols exert their anti-apoptotic effects through a multi-target network centered on the regulation of the Bcl-2/Bax/caspase-3 signaling axis, while synergistically activating antioxidant pathways (105). Under hepatotoxic conditions, bioactive components upregulate the expression of the anti-apoptotic protein Bcl-2 and downregulate the expression of the pro-apoptotic protein Bax, thereby reducing the Bax/Bcl-2 ratio. This suppresses the activation of caspase-9 and the subsequent downstream caspase-3 cascade, stabilizes the mitochondrial membrane potential, reduces programmed hepatocyte apoptosis and necrosis, and preserves the integrity of hepatic parenchymal cells (84). Concurrently, many extracts can scavenge reactive oxygen species (ROS) through the activation of the Nrf2/HO-1 antioxidant pathway. Given that oxidative stress serves as a critical upstream trigger for the intrinsic apoptotic pathway, this mechanism reduces the generation of apoptotic signals at the source (86). In addition to hepatocyte intrinsic apoptosis restraint, polyphenols mitigate inflammatory pyroptosis in immune-parenchymal cell crosstalk, limiting the release of DAMPs and halting further immune-cell recruitment to injured liver tissue.

Overall, polyphenols form a comprehensive liver-protection regulatory network that targets both parenchymal hepatocytes and diverse hepatic immune cells through multi-target cross-regulation and coordinated coupling of multiple pathways (**Figure 3**). This constitutes the core molecular basis for their more comprehensive and safer hepatoprotective advantages compared to single-target chemical drugs. It should be noted that most mechanistic observations are obtained from cell experiments and rodent models; human clinical evidence regarding polyphenol-driven liver immunomodulation remains scarce. In addition, different polyphenol subtypes exhibit distinct potency in activating or inhibiting these signaling cascades. Future translational studies should prioritize well-designed human intervention trials incorporating immunological endpoints (e.g., peripheral immune cell phenotyping and circulating cytokine panels), instead of relying exclusively on rodent-derived data.

**Figure 3:**
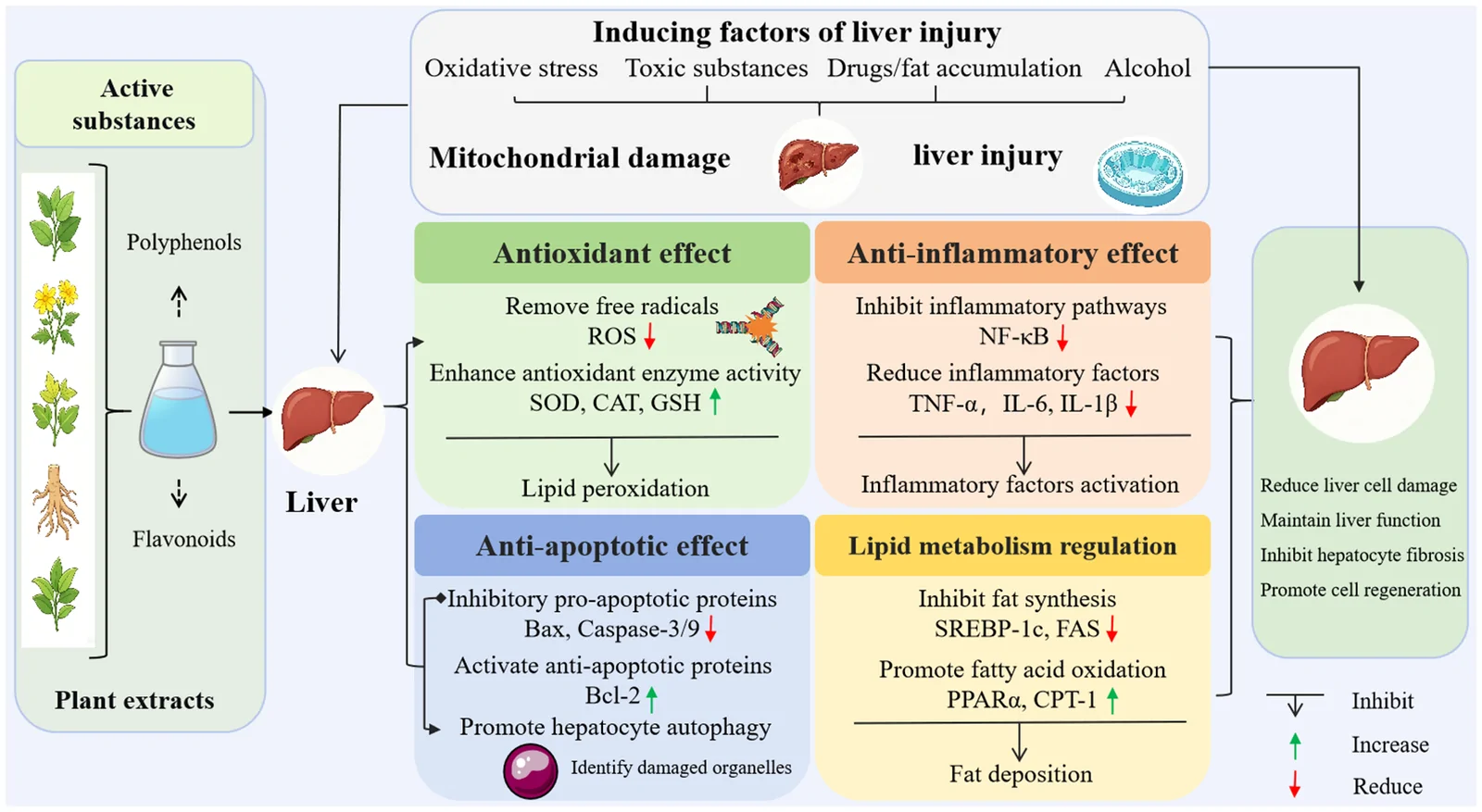
Schematic diagram summarizing the core molecular mechanisms whereby natural bioactive polyphenols exert hepatoprotective actions via multi-target signaling pathways. These protective effects act on both parenchymal hepatocytes and intrahepatic immune cell compartments.

## 5 Application limitations of polyphenols

Although polyphenols possess multi-target hepatoprotective advantages, the inherent structural characteristics of these natural compounds render free-state monomers with common problems such as poor stability, inefficient *in vivo* absorption, and undesirable sensory qualities. These issues greatly limit their functional product development and clinical translational efficacy, constituting a major bottleneck restricting their industrial application (106). Studies have shown that the antioxidant activity of polyphenols in clove extract significantly declines after 40 days of storage at room temperature or 7 days of storage at 60 °C; however, microencapsulation can effectively mitigate this activity loss (107). During spray drying of kratom leaf extract, the retention rates of total phenolics and total flavonoids ranged from 65.2% to 79.6% and 61.4% to 76.8%, respectively, while DPPH antioxidant activity decreased by approximately 20% during processing (108). Anthocyanins also face degradation risks during spray-drying microencapsulation—unencapsulated Fridericia chica extract exhibited a total phenolic loss of 88% after 180 days of storage, whereas gum arabic-encapsulated samples lost only 0.74% (109). These stability deficiencies result in severe activity loss of free-state extracts during thermal processing, sterilization, storage, and transportation, along with substantial batch-to-batch variability, making it difficult to ensure consistent hepatoprotective efficacy in final products. Therefore, strategies such as encapsulation protection and process optimization are urgently needed to address these issues.

In addition, the low oral bioavailability and insufficient liver-targeting ability of free-state natural hepatoprotective bioactive components represent core bottlenecks that limit their *in vivo* therapeutic efficacy. Most flavonoids, curcuminoids, and terpenoids possess extremely low water solubility, making them difficult to adequately dissolve and absorb after oral administration (110, 111). The strong acidic environment of the stomach, intestinal enzymatic systems, and hepatic first-pass metabolism can substantially degrade the bioactive components, resulting in extremely low concentrations of the prototype drug reaching the systemic circulation (112). Furthermore, once absorbed into the bloodstream, the lack of active liver-targeting capacity leads to nonspecific systemic distribution, preventing the attainment of effective concentrations at injured liver tissues (113).

Beyond physicochemical and *in vivo* pharmacokinetic deficiencies, most natural hepatoprotective extracts exhibit significant sensory disadvantages. Alkaloids and polyphenolic components generally possess intense bitterness, astringency, and pungent off-flavors with strong sensory irritation (114, 115). At the current stage, relying solely on formulation adjustments is insufficient to completely mask these inherent flavor defects, greatly limiting the widespread application of natural hepatoprotective extracts in daily-use hepatoprotective food products and hindering their civilian industrial popularization.

In summary, free-state natural hepatoprotective bioactive components suffer from multiple shortcomings, including poor processing stability, low oral bioavailability, weak liver-targeting ability, and undesirable sensory properties, resulting in a paradoxical situation of “excellent *in vitro* activity, insufficient *in vivo* efficacy, and difficulty in industrial translation”. To address these bottlenecks, it is urgently necessary to optimize the physicochemical properties and *in vivo* delivery efficiency of natural bioactive components through strategies such as structural modification, microencapsulation, nanodelivery, and composite stabilization technologies, thereby overcoming their application limitations. This also represents a key research direction in the current field of natural hepatoprotective products.

These drawbacks are not independent: poor processing and gastrointestinal stability further reduce oral bioavailability, while unpleasant sensory properties restrict food-product application. Therefore, single modification approaches are insufficient to resolve all above-mentioned constraints, which necessitates combined stabilization and delivery strategies illustrated in the next chapter. Moreover, most existing *in vivo* efficacy assessments only detect serum transaminases and antioxidant indicators, lacking systematic immune-phenotyping of intrahepatic immune-cell subsets, inflammasome activation status, and gut-liver immune homeostasis, which hinders full interpretation of polyphenol pharmacological outputs.

## 6 Stabilization modification technologies for polyphenols

Based on the core bottlenecks summarized in Chapter 5 for free-state natural hepatoprotective bioactive components—namely, poor processing stability, low oral bioavailability, insufficient targeting ability, and sensory defects—various stabilization modification and precision delivery technologies have emerged as key approaches to resolve the paradox of “high *in vitro* activity, low *in vivo* efficacy, and difficulty in industrial translation” of natural products. This chapter systematically reviews the mechanisms of action and technical advantages of various stabilization and delivery technologies in light of the latest domestic and international research progress (**Figure 4**), aiming to provide systematic theoretical support and technical reference for the high-end development, industrial translation, and clinical application of natural hepatoprotective functional formulations.

**Figure 4:**
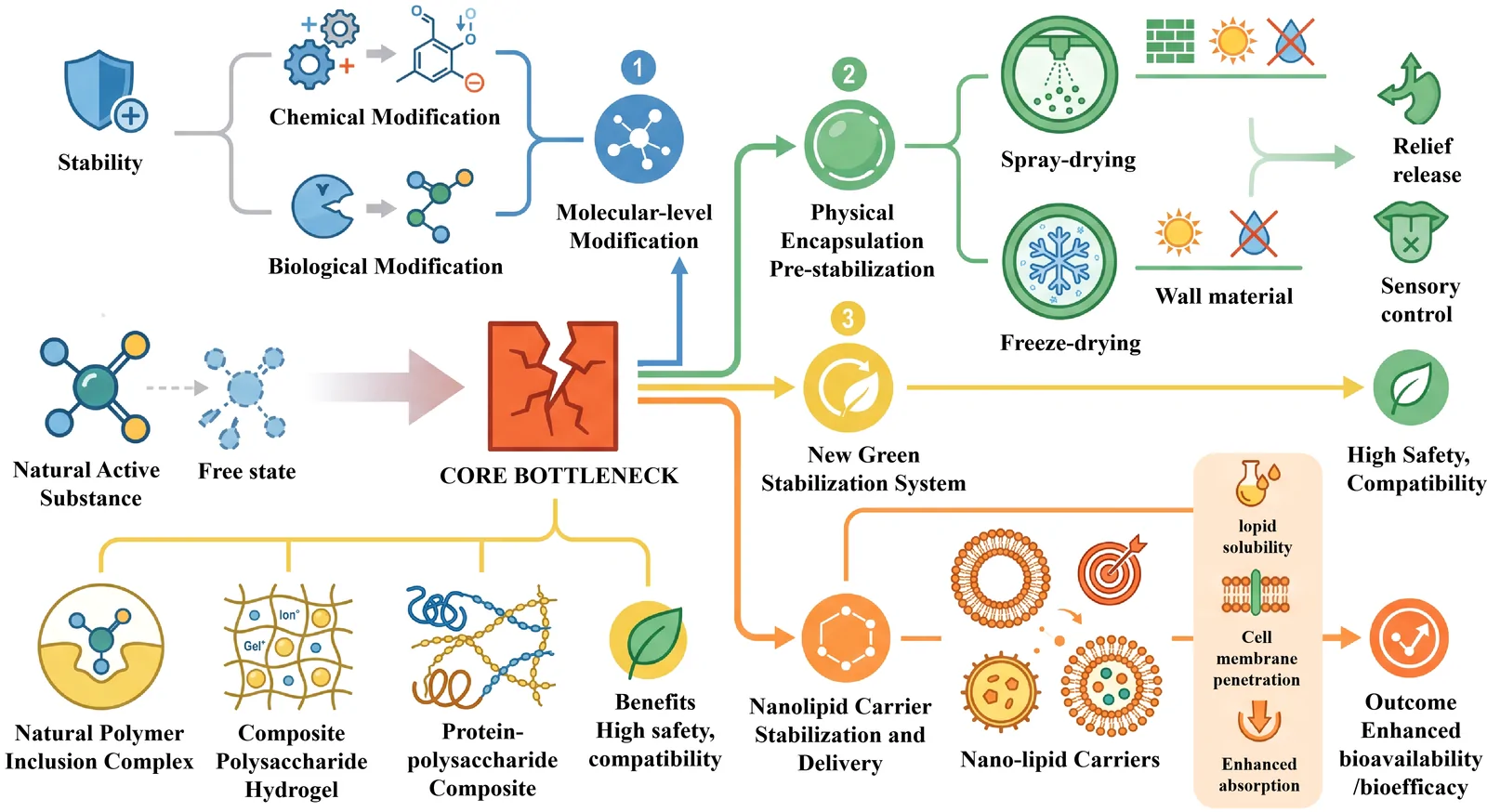
It illustrates the core stability-related bottlenecks of free polyphenols, as well as diverse delivery strategies for improving their stability and bioavailability.

### 6.1 Molecular-level stabilization modification technologies

Molecular-level stabilization modification represents a core technology that fundamentally optimizes the physicochemical properties and pharmacological activities of natural hepatoprotective components, and can be primarily categorized into chemical modification and biological modification. These approaches can effectively address the inherent shortcomings of bioactive components, including susceptibility to oxidation, poor water solubility, and metabolic instability. Chemical modification enhances the stability of bioactive components through structural modification methods such as prenylated, sulfation and carboxymethylation. Studies have shown that a series of prenylated resveratrol derivatives exhibit favorable antioxidant activities and can serve as potential lead compounds. Furthermore, these derivatives display no neurotoxicity and possess stronger inhibitory capacity against oxidative stress in P19-derived neuronal cells (116). A previous study reported the fabrication of succinylated chitosan (SCs) nanoparticles for polyphenol encapsulation via ionic cross-linking. Chitosan (Cs) was succinylated by the reaction between its -NH_2_ groups and succinic anhydride. The results demonstrated that the encapsulation efficiency of gallic acid reached 88 %, whereas that of epigallocatechin-3-gallate was 65 % and propyl gallate was only 27 % (117). Biological modification employs techniques such as enzymatic modification to achieve structural optimization of bioactive components under mild conditions. A study has shown that oral administration of enzymatically modified citrus flavonoids significantly suppressed alcohol-induced hepatic injury by maintaining malondialdehyde, GSH, and catalase levels near normal, while also inhibiting the abrupt increases in proinflammatory cytokines (IκB-α, TNF-α, IL-1β, IL-6) caused by chronic alcohol uptake (118). Furthermore, studies have demonstrated that cyclodextrin glucanotransferase-catalyzed glycosylation of rutin yields α-monoglucosyl rutin (α-GR) with markedly improved water solubility. In a cyclophosphamide-induced murine liver injury model, α-GR significantly reduced serum AST, ALT, and TBA levels, modulated antioxidant enzyme activities, and suppressed inflammatory cytokine expression, thereby effectively attenuating hepatic damage (119).

*Advantages and limitations:* Molecular chemical-enzymatic modification fundamentally optimizes intrinsic physicochemical properties of polyphenols. Chemical modification can greatly improve stability and solubility, yet it may generate unknown by-products and raises food-safety concerns. Enzymatic modification proceeds under mild conditions with higher safety, but suffers from high-cost enzymes and difficulties for large-scale industrial production.

### 6.2 Physical pre-stabilization encapsulation technologies

Physical pre-stabilization technologies do not alter the molecular structure of active components. Instead, they construct external protective barriers for free active components through physical encapsulation and environmental conditioning. The wall materials can form dense physical protective layers that shield against adverse external factors such as high temperature, light, oxygen, and pH extremes. Simultaneously, they can completely encapsulate bitter and off-flavor substances, enable sustained release of active components, and protect against gastric acid degradation (120). This technology substantially enhances the processing and storage stability of active components, and offers the advantages of high safety, mature processing techniques, and suitability for industrial-scale production. Among these, traditional microencapsulation technologies, exemplified by spray-dried microcapsules and freeze-dried microcapsules, are the most widely applied. A study reported chitosan nanoparticles loaded with litchi polyphenols (LP-CSNPs) fabricated by ionic gelation. Experimental results revealed that the system achieved an encapsulation efficiency of 45.53 % for polyphenols. After 100 h incubation in simulated gastric fluid (pH 1.2) and simulated intestinal fluid (pH 6.8), the release rates were 58.34 % and 81.68 %, respectively. Compared with free litchi polyphenols, LP-CSNPs could effectively enhance storage stability (121). Guo et al. (2020) prepared curcumin microparticles using spray drying and freeze drying, and found that the microencapsulated curcumin exhibited significantly enhanced stability under high-temperature and acidic conditions compared to free curcumin (122).

*Advantages and limitations:* Physical microencapsulation possesses high safety and mature manufacturing routes suitable for industrial scale-up, which effectively shields polyphenols from heat, light and oxygen and masks off-flavor. Nevertheless, conventional microcapsules lack active liver-targeting capability and cannot substantially enhance intracellular membrane penetration. Additionally, few studies have evaluated whether wall-material components themselves trigger unintended hepatic or intestinal immune-cell activation.

### 6.3 Novel green stabilization systems

The “green” characteristic of the novel green stabilization system described herein indicates that the system is fabricated from food-grade starting materials, non-toxic crosslinking agents and biodegradable constituents without harmful chemical additives. In recent years, novel green stabilization technologies based on natural polymers and biocompatible excipients have developed rapidly (134). These technologies offer the advantages of zero additives, high safety, strong stabilization effects, and broad adaptability, making them an important alternative to traditional modification approaches (123). Natural polymer materials possess special structural features, such as externally hydrophilic and internally hydrophobic cavities, which enable the specific inclusion of hydrophobic hepatoprotective active molecules through intermolecular forces to form supramolecular inclusion complexes. A study evaluated the hepatoprotective effects of native San Huang Shel Shin Tang (SHSST) and β-cyclodextrin-modified SHSST complex (SHSSTc) in rats with carbon tetrachloride (CCl_4_)-induced acute hepatotoxicity. Compared with native SHSST, SHSSTc (40 mg/kg/day) significantly downregulated the CCl_4_-induced protein expression of TGF-β and caspase-8 (124). Another study has also shown that curcumin/cyclodextrin polymer inclusion complexes (CUR/CDP) can inhibit apoptosis by downregulating the expression of p-p53 and caspase-3, thereby exerting a significant protective effect against H_2_O_2_-induced oxidative damage in LO2 cells (125). Bao et al. (2024) employed hydroxypropyl methylcellulose (HPMC) as the film-forming matrix, and combined three sulfated polysaccharides including chondroitin sulfate (CS) with blueberry anthocyanins (BA) to fabricate oral film formulations. The results indicated that the incorporation of sulfated polysaccharides markedly improved the stability of BA. After 8-day accelerated storage under light exposure, the BA retention rate of the CS-BA/HPMC system was 5.5-fold higher than that of the control group, exhibiting the optimal stabilizing performance (126). Protein–polysaccharide composite green modification systems, on the other hand, form a composite network through hydrophobic binding of proteins and cross-linking of polysaccharides, simultaneously enhancing stability, emulsifying properties, and bioavailability. A study found that silymarin-loaded albumin-hyaluronic acid nanoparticles alleviated CCl_4_-induced liver fibrosis by inhibiting the TGF-β/Smad-3 pathway (downregulating Collagen I, TGF-β1, SMAD3), while activating hepatic progenitor stem cells (upregulating HNF1β, FOXl1, CD90, Vimentin, and CD105) to promote liver regeneration, without inducing inflammation, and showed superior efficacy over free silymarin (89).

*Advantages and limitations:* Green supramolecular systems based on natural polymers feature good biocompatibility and food-grade compatibility. However, such supramolecular inclusion complexes usually exhibit limited drug-loading capacity, and their performance is highly sensitive to environmental pH and temperature variations.

### 6.4 Nanostructured lipid carriers for stabilization and delivery

Nanostructured lipid carriers represent a core technology that overcomes the bottleneck of traditional stabilization techniques, which “only maintain stability without enhancing efficacy”. They combine multiple advantages, including physical stabilization protection, high loading capacity, membrane penetration capability, and biocompatibility. These carriers can specifically address the challenges of poor water solubility, gastrointestinal instability, and difficulty in membrane absorption faced by natural hepatoprotective components, serving as a key bridge between stabilization and efficient *in vivo* delivery (127, 128). Solid lipid nanoparticles (SLNs) efficiently encapsulate hydrophobic hepatoprotective agents via a solid lipid matrix that locks molecules and enhances stability. Bee pollen extract-loaded SLNs (118.6 nm, 92.7% EE) showed 48-hour sustained release, significantly reducing ALT, AST, ALP, and MDA while boosting antioxidant enzymes and alleviating hepatic pathology in lead acetate-induced rat liver injury (129). Liposomes, possessing a biomimetic phospholipid bilayer structure, can co-encapsulate both hydrophilic and hydrophobic components. Catalase-modified silymarin liposomes (CAT-LP) exert synergistic anti-fibrotic effects via catalase-mediated ROS scavenging and silymarin-mediated inhibition of hepatic stellate cell (HSC) activation. In a CCl_4_-induced mouse model, CAT-LP reduced MDA and transaminase levels, increased SOD activity, and attenuated collagen deposition (130). Polymer-lipid hybrid nanoparticles (PLN) combine the stability of polymers with the high drug-loading capacity of lipids. Green coffee extract-loaded, with an encapsulation efficiency of 55%, achieved sustained release for 24 hours and downregulated the expression of lipid accumulation and fibrosis-related genes, including PLIN1 and ACTA2, in HepG2 and HHSC-N cells (131).

*Advantages and limitations:* Nanostructured lipid carriers combine stabilization, high loading capacity and improved cellular permeability. Many prototypes exhibit promising liver-protective effects in animal models. Still, complicated preparation processes and high cost severely hinder their transformation into real food-grade products, and most studies remain confined to laboratory scale. Besides, the immunological impact of nanocarriers on Kupffer-cell activation and liver immune microenvironment is frequently overlooked in food-oriented research.

At present, single technologies still have certain limitations. The core development trend in the stabilization and delivery technologies for natural hepatoprotective extracts lies in the integrated application of multiple technologies—namely, an integrated technical system combining “molecular modification - green stabilization - nanocarriers - intelligent targeting”. This integrated approach simultaneously addresses processing stability, sensory quality, oral absorption efficiency, liver-targeting capability, medication safety, and modulates local hepatic immune microenvironment, thereby thoroughly resolving the core bottlenecks hindering the industrial production and clinical translation of natural hepatoprotective products.

## 7 Current research bottlenecks and future development trends

A substantial body of research has demonstrated that natural bioactive substances such as flavonoids and polyphenols possess hepatoprotective effects. However, the overall research landscape still faces key bottlenecks, including fragmented mechanistic studies, disconnection between research and technological translation, and inadequate evaluation systems. Importantly, critical gaps persist in immunological dimensions. The majority of animal studies rely merely on circulating pro-inflammatory cytokine quantification, lacking high-dimensional profiling of intrahepatic immune-cell repertoires. Whether polyphenols directly act on hepatocytes or indirectly remodel liver immunity via gut-liver immune crosstalk remains poorly dissected. In addition, the dose-dependent hormetic effects of polyphenols on innate-adaptive immune compartments have not been sufficiently characterized. Notably, current polyphenol research also presents a prominent academic bias that overemphasizes hepatoprotective efficacy while neglecting systematic toxicological assessment. Although dietary polyphenols are generally safe at conventional dietary intake levels, their dose-dependent biological duality cannot be ignored: high-dose supplementation and fortified functional food applications may trigger pro-oxidant responses and potential endocrine disruption, yet unified toxicological threshold standards and long-term safety verification remain absent. Such ambiguous risk-benefit balance further hinders the commercialization of high-end hepatoprotective functional products and their clinical popularization. Currently, research on natural hepatoprotective extracts still exhibits significant systemic deficiencies. For instance, stability studies are mostly confined to evaluations under *in vitro* processing conditions, neglecting the dynamic activity loss caused by gastrointestinal metabolism and the hepatic microenvironment *in vivo*, resulting in a disconnection between *in vitro* stability data and *in vivo* efficacy. High-end targeted delivery systems generally face barriers to food-grade translation; most intelligent nanocarriers are costly and poorly processable, making them unsuitable for large-scale production of functional foods and thus remaining confined to fundamental laboratory research. Meanwhile, existing studies commonly lack a quantitative coupling analysis of “delivery efficiency–intrahepatic active concentration–hepatoprotective efficacy”, and no unified correlative evaluation system has been established, which constrains the precise optimization of delivery systems and in-depth mechanistic exploration. Moreover, most studies utilize crude polyphenol extracts rather than pure monomers; batch-to-batch variation of plant extracts contributes to inconsistent biological efficacy, which represents another major barrier for industrial translation. In addition, gut-liver axis effects are frequently overlooked: many polyphenols exert bioactivities after metabolic transformation by gut microbiota, which should be considered when designing delivery carriers.

In response to the aforementioned bottlenecks, including unresolved toxicological ambiguities, future research and product development must move beyond traditional single-modification approaches and establish an integrated innovation system. (1) Mechanistic research: Construct multi-dimensional dynamic evaluation models to quantitatively correlate delivery performance, intrahepatic polyphenol accumulation and hepatoprotective efficacy; prioritize immunological readouts including inflammasome activation status, macrophage polarization and T-cell subset profiling; pay more attention to gut-liver-axis mediated polyphenol metabolism and gut-associated immune responses, clarify the dual regulatory mechanisms underlying their dose-dependent efficacy and toxicity; (2) Technological translation: Develop low-cost, food-grade green carriers; adapt intelligent nanodelivery concepts from pharmaceutical research to food-industry requirements; (3) Evaluation system: Establish unified quantitative efficacy traceability and toxicological safety assessment systems to reduce the disconnect between *in-vitro* results and *in-vivo* outcomes, and to achieve precise balance between maximal hepatoprotective benefits and minimal toxic risks.

## 8 Conclusion

In summary, natural polyphenol extracts, owing to their unique advantages of multi-target regulation, low toxicity, high safety, and suitability for dietary intervention, demonstrate broad application prospects and immense industrial potential in the development of novel hepatoprotective functional formulations. Nevertheless, their dose-dependent toxicological risks, unclear safe dosage windows, and insufficient long-term safety evidence currently restrict their large-scale application in high-dose functional fortification. In view of the deficiencies in the current research framework, future efforts should focus on multidimensional technological innovation, toxicological risk clarification, and translational implementation to forge a full-chain development pathway spanning “fundamental research–pilot scale-up–clinical translation”. Collectively, this review constructs an integrated framework spanning toxicological characteristics, material basis, multi-target molecular mechanisms, stabilization-delivery approaches, and translational obstacles, providing theoretical insights for developing polyphenol-based hepatoprotective functional foods and dietary intervention strategies. It should be emphasized that polyphenol-enriched functional food cannot replace clinical pharmaceutical interventions for liver diseases; all dietary-intervention conclusions are derived from pre-clinical evidence, and human immunological data are urgently required for future clinical validation.
